# Possible predisposition for colorectal carcinogenesis due to altered gene expressions in normal appearing mucosa from patients with colorectal neoplasia

**DOI:** 10.1186/s12885-019-5833-8

**Published:** 2019-06-28

**Authors:** Christian Hunnicke Petersen, Badar Mahmood, Christoffer Badsted, Tina Dahlby, Hanne Borger Rasmussen, Mark Berner Hansen, Niels Bindslev

**Affiliations:** 1 Digestive Disease Center K, Bispebjerg Hospital, DK-2400 Copenhagen, Denmark; 2 Department of Biomedical Sciences, Faculty of Health Sciences, University of Copenhagen, DK-2200 Copenhagen, Denmark

**Keywords:** Extracellular signal-regulated kinase (ERK), Akt, Prostaglandin E2, β-Catenin, Colorectal cancer

## Abstract

**Background:**

Investigations of colorectal carcinogenesis have mainly focused on examining neoplastic tissue. With our aim of identifying potentially cancer-predisposing molecular compositions, we chose a different approach by examining endoscopically normal appearing colonic mucosa of patients with and without colorectal neoplasia (CRN). Directed by this focus, we selected 18 genes that were previously found with altered expression in colorectal cancer affected mucosa.

**Methods:**

Biopsies of colonic mucosa were sampled from 27 patients referred for colonoscopy on suspicion of colorectal disease. Of these, 14 patients had present or previous CRN and the remaining 13 patients served as controls. Using qPCR and Western blot technique, we investigated mRNA and protein expressions. Expressions were investigated for selected kinases in the extracellular signal-regulated kinase/mitogen activated protein kinase (ERK/MAPK), the phosphoinositide 3-kinase/Akt, and the Wnt/β-catenin pathways as well as for selected phosphatases and several entities associated with prostaglandin E2 (PGE_2_) signaling. Colonic mucosal contents of PGE_2_ and PGE_2_ metabolites were determined by use of ELISA.

**Results:**

We found up-regulation of *ERK1*, *ERK2*, *Akt1*, *Akt2*, *PLA2G4A*, prostanoid receptor *EP3* and phosphatase scaffold subunit *PPP2R1B* mRNA expression in normal appearing colonic mucosa of CRN patients compared to controls.

**Conclusion:**

Present study supports that even normal appearing mucosa of CRN patients differs from that of non-CRN patients at a molecular level. Especially expression of *ERK1* mRNA was increased (*p* = 0.007) in CRN group. *ERK1* may therefore be considered a potential candidate gene as predictive biomarker for developing CRN. Further validation in larger cohorts are required to determine such predictive use in translational medicine and clinics.

## Background

### Investigating endoscopically normal appearing mucosa

Most investigations of tumorigenesis focus on alterations in neoplastic tissues or transformed cells in culture. Due to progressive derangement in neoplastic tissue of the genome, growth factor-signaling, and metabolic networks, screening of colorectal neoplasia (CRN) tissue itself demonstrates many alterations. Thus, investigations on CRN tissues provide limited information regarding possible predisposing factors and their role in transforming normal appearing mucosa to CRN. Screening of endoscopically normal appearing mucosa from patients with CRN and comparing it to mucosa of non-CRN patients may generate important new insights into identifying subjects with increased risk of developing CRN (risk stratification, predictive biomarker). As such, we and a few others have communicated altered expression of several genes and proteins in normal appearing mucosa from CRN patients [1–4].

### Wnt/β-catenin-, ERK/MAPK- and PI3K/Akt pathways in colorectal cancer

Among the frequently disrupted signaling pathways, perturbation of the Wnt/β-catenin pathway has been identified in early lesions of colorectal epithelium. Concurrently, Wnt/β-catenin perturbation is regarded a major initiating event in development of CRN as its tumor-suppressor gene adenomatous polyposis coli (APC) is inactivated in about 80% of sporadic colorectal cancers, CRC. APC inactivation leads to increased nuclear transfer of β-catenin with formation of a constitutively active β-catenin-T-cell factor complex [5]. In addition to perturbation of Wnt/β-catenin pathway, two other major pathways, the extracellular signal-regulated kinase/mitogen activated protein kinase (ERK/MAPK) and phosphoinositide 3-kinase/Akt (PI3K/Akt)-signaling pathways, are frequently overactive in CRC. Activation of these pathways stimulate cell growth, proliferation and survival [6, 7]. As such, constitutive activation of the ERK/MAPK pathway occurs via mutations in KRAS in about 50% of CRCs [8]. Less frequently, dysregulation of PI3K/Akt pathway is observed following mutations in the CA subtype of the phosphoinositide-3-kinase gene (PIK3CA) or in the PI3K/Akt-related phosphatase and tensin homolog (PTEN) [9, 10].

### Prostaglandin E2 in colorectal carcinogenesis

**Fig. 1:**
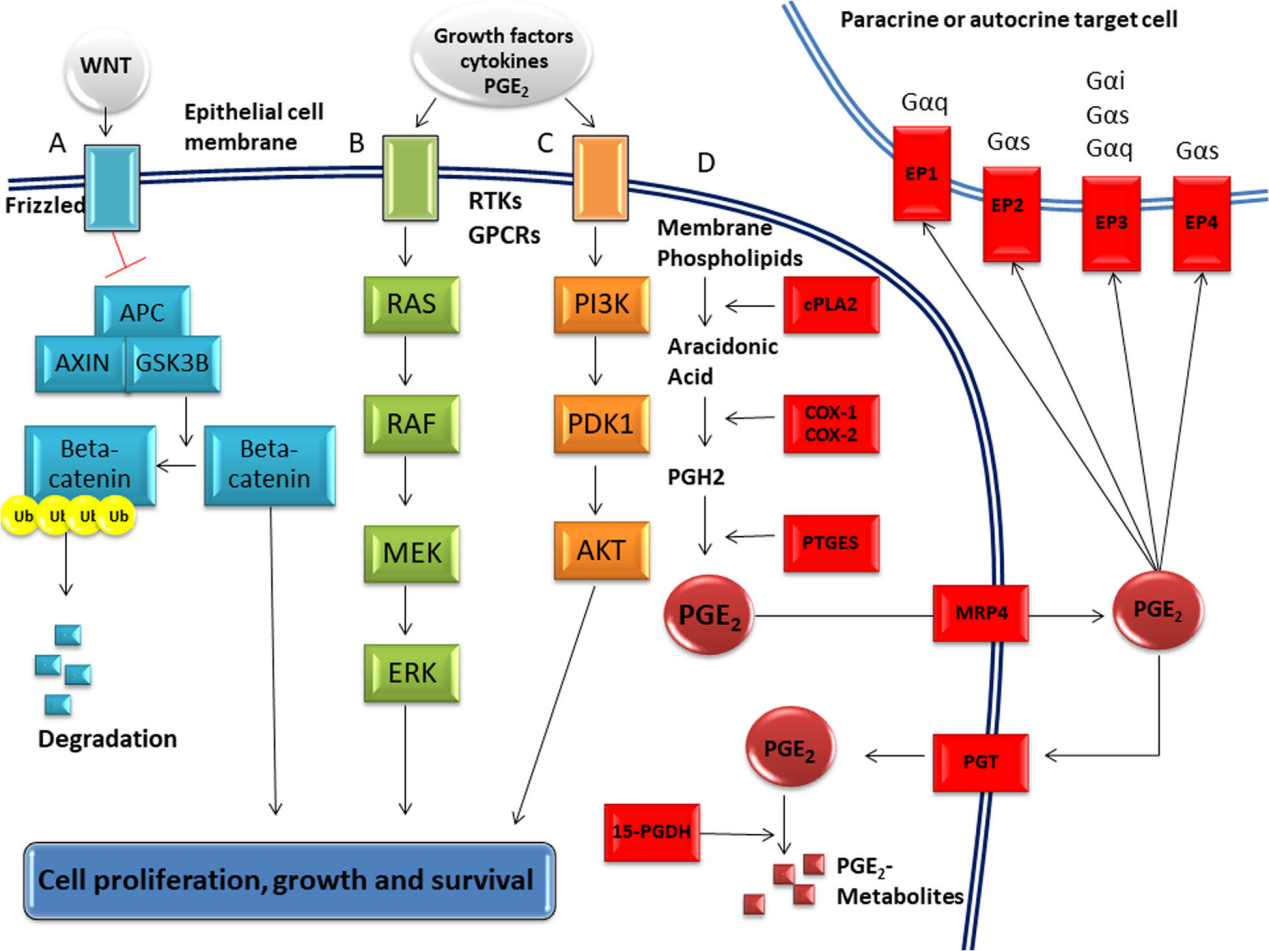
Simplified schematic illustration of pathways for Wnt/β-catenin, ERK/MAPK and PI3K/Akt and PGE_2_-metabolism. A) Canonical Wnt/β-catenin signaling. The engagement of the Wnt receptor, Frizzled, leads to the inhibition of the β-catenin destruction complex, composed of APC, axin and GSK3β. β-catenin thereby avoids ubiquitination and subsequent degradation, thus allowing it to translocate to the nucleus to activate an array of regulatory genes. B) The RAS/RAF/MEK/ERK MAPK pathway. Stimulation of the receptor tyrosine kinase (RTK) or G-protein coupled receptors (GPCRs) leads to sequential activation of RAS, RAF, MEK, and ERK causing modification of substrates promoting cell survival and proliferation. C) In the PI3K/Akt pathway, activation of the RTK or GPCRs leads to sequential modification of phosphatidyl inositol residues of the phospholipid bilayer. In this process, PI3K generates PIP3. PIP3 in association with PDK1 activates Akt. Akt then modulates the activity of downstream substrates including mTOR, thus promoting proliferation and cell survival. D) PGE_2_-metabolism. PGE_2_-synthesis begins with catalytic hydrolysis of membrane phospholipids by cytoplasmic phospholipase A2 (cPLA_2_), thus releasing arachidonic acid (AA). By the action of the COX-1 and COX-2, AA is converted to prostaglandin H_2_ (PGH_2_). PGH_2_ is then converted to PGE_2_ by prostaglandin E synthase (PTGES). The main exporter of PGE_2_ is thought to be multi-drug resistance related polypeptide 4 (MRP4). Removal of PGE_2_ from the extracellular compartment around target cells occurs by diffusion to the blood stream and subsequent uptake and degradation in lung, liver or kidney endothelial cells or by import to colonic epithelial cells through the prostaglandin transporter (PGT) and subsequent degradation by 15-prostaglandin dehydrogenase (15-PGDH). Through autocrine and paracrine signaling, extracellular PGE_2_ stimulates the prostaglandin receptors EP1–4. The EPs are GPCRs with EP1 being Gαq-coupled while EP2 and EP4 are Gαs-coupled. EP3 is capable of coupling with different G-proteins including Gαi, Gαs and Gαq

During carcinogenesis the regulatory system of cell cycle is affected by both internal genetic alterations and external signaling. In particular, cytokines, growth factors and eicosanoids trigger signal transduction cascades through receptor tyrosine kinases and G protein-coupled receptors e.g., epidermal growth factor via its receptor EGF and PGE_2_ through prostanoid receptors type 1–4. Among the prostanoids, prostaglandin E_2_ (PGE_2_) has been identified as the principal entity promoting cell growth and survival in CRN. It is believed that PGE_2_ execute these effects via the PI3K/Akt-, ERK/MAPK- and Wnt/β-catenin pathways [11]. Induction of PGE_2_ signaling is also well-established as an early and critical step in development of CRN and in tumor progression [12, 13]. All these pathways and PGE_2_ metabolism are schematized in Fig. 1.

### Hypothesis and aim of study

We hypothesized that up-regulation of some specific CRC-associated kinases and PGE_2_-related proteins occur in normal appearing colonic mucosa. The aim of this study was to test this 0-hypothesis by screening colonic mucosa for a panel of 18 specific genes, all proven altered and involved in CRC development. Tissue samples were endoscopic biopsies obtained from patients with and without CRN. Indeed, identifying perturbation of some of these signaling networks might give further insight into colorectal carcinogenesis per se and also identify potential predictive biomarkers.

## Materials and methods

### Study population

Patients ≥ 50 years of age, referred for colonoscopy on suspicion for colorectal disease, were screened for enrollment in the study. Patients with a history of CRN, or CRN detected during colonoscopy, were included in CRN group. Patients with no present or history of CRN represents the control group. Patients were excluded if they received weekly medications in the form of nonsteroidal anti-inflammatory drugs, paracetamol, systemic corticosteroids and/or cytostatics. Furthermore, incomplete colonoscopy, inflammatory bowel disease, malabsorption or previous sigmoid resection and exposure to radiation or chemotherapy within the last year precluded enrollment. Finally, patients were also excluded if diagnosed with any of the genetic CRN syndromes (e.g. adenomatous polyposis coli and hereditary nonpolyposis colorectal cancer), as the focus of this study was sporadic CRN. Risk stratification of adenomas was performed based on number, size and histology grade in accordance with the European guidelines for quality assurance in CRC screening and diagnosis [14].

### Biopsy procedure

Six biopsies from each patient were obtained during endoscopy from endoscopically normal appearing mucosa using standard biopsy forceps (Boston Scientific, Radial Jaw 4, outside diameter of 2.2 mm). Biopsies were obtained approximately 30 cm orally from the anal verge on retraction of the endoscope and at least 10 cm from endoscopically abnormal appearing mucosa.

### Statistical analysis

Values were expressed as the mean ± SEM and as fold change. Data was analyzed using two-tailed unpaired t-test. A *p-*value less than 0.05 was considered statistically significant. Bonferroni correction was applied. Calculations were performed using Microsoft Excel 2007 and GraphPad Prism 6.01.

### qPCR

Two biopsies, obtained from each patient, were stored in RNA*later* (Thermo Fisher Scientific, Wilmington, DE, USA) at − 80 °C. Biopsies were homogenized using a TissueLyser II (Qiagen, Copenhagen, Denmark), and RNA was extracted using RNeasy Mini Kit (Qiagen, Copenhagen, Denmark). RNA concentration and purity were determined using a NanoDrop® 2000 (Thermo Fisher Scientific, Wilmington, DE, USA), the latter by A_260_/A_280_ and A_260_/A_230_ absorbance ratios. RNA was converted to cDNA using the iScript™ cDNA Synthesis Kit (BioRad, Copenhagen, Denmark) according to the manufacturers protocol. Primers against genes of interest were designed, synthesized and quality controlled by Primerdesign Ltd. (Primerdesign Ltd., Chandler’s Ford, UK). Primers against β-actin were designed and synthesized by Section of Endocrinology Research, Department of Biomedical Sciences, University of Copenhagen, primerbank reference 4501885a1. Primer sequences are listed in Table 1. cDNA was amplified on a 7900HT Fast Real-Time PCR System (Applied Biosystems, Foster City, CA, USA) using Fast SYBR® Green Master Mix (Applied Biosystems) in accordance with the manufacturers protocol. All samples were run in triplicate with β-actin primers on all plates. Results were analyzed using SDS 2.4 (Applied Biosystems), and expression was calculated by the 2^-ΔCt^ method.

**Table 1:** Quantitative real-time PCR primer sequences

Target	Forward 5’ → 3’	Reverse 5’ → 3’
*β-actin*	CATGTACGTTGCTATCCAGGC	CTCCTTAATGTCACGCACGAT
*PLA2G4A*	GAAAAGTGGGCTAAAATGAACAAG	GGGCAATCTTTCTCCATATCAG
*COX1*	CGTGTGTGTGACCTGCTGAA	GTACTCCTCGATGACAATCTTGATG
*COX2*	CAGGCTTCCATTGACCAGAG	TTTCTCCTGTAAGTTCTTCAAATGAT
*PTGES*	GTGGCTATACCTGGGGACTT	AATCCAAGGGGCTAAGAAACAT
*EP1*	ATCTGCTGGAGCCCAATGC	GATCTGGTTCCAGGAGGCAA
*EP2*	CAGACCCTGGTGGCACTG	CGAAGAGCATGAGCATCGTG
*EP3*	TCAATCAGACATCAGTTGAGCAC	TTTCTTAACAGCAGGTAAACCCAA
*EP4*	AGTTTGGAGCGAGAAGTCAGTA	GCGGCAGAAGAGGCATTTG
*15-PGDH*	AGTAGTGAACATCAATGAACATCTGA	ACTGGGCAAACCGACATCAT
*PGT*	GCCACAGCAGATGAAGCAAG	CCACCAGGACGAAGAGTGAG
*ERK1*	AGAGATCATGCTGAACTCCAAG	GTGCTTGCCAGGGAAGATG
*ERK2*	TGGATGTGGTGTTATGGAAAGA	AAGCAGAGACGCAGAATGAC
*AKT1*	GGCACCCCTTCCTCACAG	GGCGTACTCCATGACAAAGC
*AKT2*	CTGCGGAAGGAAGTCATCATT	GGTCGTGGGTCTGGAAGG
*β-catenin*	CCATTACAACTCTCCACAACCT	GAGCAAGGCAACCATTTTCTG
*GSK3B*	AAGTAATCCACCTCTGGCTACC	AGAAGCAGCATTATTGGTCTGTC
*PPP2R1B*	GGTTCACCTCTCGCACATCT	TCTGAGCACAAGGAACGGAAT
*PTPRM*	ACTCGTTGCCACAGTTATAATCTC	GTGTATGGTGACAGGTTAGTGATC

Left column: Names of all qPCR-analysed targets. Middle column: Forward primer sequences written 5′ → 3′. Right column: reverse primer sequences written 5′ → 3′.

### Western blot and immunohistochemistry

#### Primary antibodies for Western blots and immunohistochemistry

The following antibodies were employed to detect target antigens by Western blot and immunohistochemistry (IHC), in parenthesis (antibody designator, dilution): goat anti-COX-1 (C-20, 1:800), mouse anti-β -catenin (E-5, 1:200) and mouse anti-PGDH (H-3, 1:250) were all purchased from Santa Cruz Biotechnology, Heidelberg, Germany. Rabbit anti-ERK1/2 (9102S, 1:1000), rabbit anti-pERK1/2 (9101S, 11,000) and rabbit anti-panAkt (C67E7, 1:1500) were all from Cell Signaling, Leiden, The Netherlands. Rabbit anti-COX-2 (SP21, 1:500) was from Spring Biosciences, Pleasanton, CA, USA. We did not succeed in detecting EP3, neither in Western blots nor in IHC staining, despite using four different antibodies. The following antibodies were tested: Mouse anti-EP3 antibody (5F5), sc-57,105 (Santa Cruz Biotechnology), Rabbit anti-EP3 antibody, 101,760 (Cayman Chemicals, Ann Arbor, MI, USA), Rabbit anti-EP3 antibody, APR-065 (Alomone Labs, Jerusalem, Israel), Rabbit anti-EP3 antibody (PTGER3), NBP1–84835 (Bio-techne, Abingdon, United Kingdom). In addition, attempts to detect cytoplasmic phospholipase A2 alpha (cPLA2A) using two different antibodies (Santa Cruz Biotechnology, sc-454 and Cell Signaling 2832) and protein phosphatase 2 isoform beta of scaffold subunit A (PPP2R1B) using mouse anti-PPP2R1B (Santa Cruz Biotechnology, sc-13,600) proved unsuccessful in both Western blots and IHC. IHC staining for PGT and for COX-2 with two different antibodies (Cayman Chemicals, 160,126 and Spring Biosciences SP21) was attempted, but was unsuccessful.

#### Homogenization and solubilization of colonic biopsies for Western blot analysis

Snap-frozen biopsies were homogenized for 20 s at 5000 rpm in 150 μl buffer (50 mM TrisHCl, pH 8.5, 5 mM EDTA, 150 mM NaCl, 10 mM KCl, 1% Triton X-100, 5 mM NaF, 5 mM β-glycerophosphate, 1 mM sodium-orthovanadate and Complete Protease Inhibitor Cocktail Tablet (Roche, Hvidovre, Denmark)) using a PreCellys 24 (Bertin Instruments, Montigny-le-Bretonneux, France) and ceramic beads (mix of 1.4 mm and 2.8 mm beads, VWR, Soeborg, Denmark). Solubilization was continued for 2 h in the same buffer at 4 °C with rotation. The solubilized samples were spun for 15 min at 15000 g and the resulting supernatant collected for further protein determination and Western blot analysis.

#### Western blotting

Twenty-four biopsies were analyzed by Western blotting (12 CRN, 12 controls). 12.5 μg protein was separated on 4–20% gradient mini-PROTEAN® TGX™ Gels (Bio-Rad Laboratories, Copenhagen, Denmark) using the Bio-Rad Laboratories minigel system. Proteins were transferred onto an Immobilon-FL Transfer Membrane 45 μM (Merck Millipore, Hellerup, Denmark) in 25 mM Tris base, 200 mM glycine, 20% methanol, 1% SDS using the same system. After transfer, membranes were stained with REVERT™ Total Protein Stain (LI-COR Biosciences, Cambridge, United Kingdom) and the staining captured using the Odyssey CLx Imaging System (LI-COR Biosciences) to adjust for protein loading (internal loading control). Membranes were subsequently blocked for 1 h at room temperature in blocking buffer (Odyssey Blocking Buffer (PBS), LI-COR Biosciences). Primary antibody was applied overnight at 4 °C in blocking buffer. IRDye® 800CW Secondary Antibodies (1:10000 dilution in blocking buffer, LI-COR Biosciences) were then applied for 30 min at room temperature and bound antibody detected with the Odyssey CLx Imaging System (LI-COR Biosciences). Western blot was repeated twice on the same samples to ensure reproducibility. Signals originating from target proteins were quantified in Image Studio Version 3.1 using REVERT™ Total Protein Stain as internal loading control. The target protein signal in each lane was normalized to the total protein stain in the corresponding lane. The normalized mean target protein signal originating from the control group was then set to 1 and all values expressed relative to this value.

#### Immunohistochemistry

Biopsies were fixed immediately in 4% formaldehyde and kept in the formaldehyde solution at 4 °C until cutting. Cryoprotection was performed by two successive overnight incubations at 4 °C in PBS containing first 20% sucrose and subsequently 30% sucrose. After cryoprotection, 10–15 μm cryostat sections were cut. For staining, sections were subjected to antigen retrieval by boiling in 10 mM citric acid pH 6.0 for 5–10 min. After washing in PBS, unspecific binding was blocked for 30 min in PBS containing 0.1% triton X-100 and 0.2% fish skin gelatine. Primary antibodies were diluted in the same buffer and incubated overnight at 4 °C. Bound primary antibody was detected by incubation in AlexaFluor®-conjugated secondary antibodies (Thermo Fisher Scientific, Roskilde, Denmark) for 1 h at room temperature. Nuclei were detected using 4′,6-diamidino-2-phenylindole (DAPI, Thermo Fisher). Sections were mounted in Prolong Diamond (Thermo Fisher Scientific). IHC staining for cytoplasmic phospholipase A2 alpha (cPLA2A), cyclooxygenase 1 and 2 (COX-1, COX-2), prostaglandin transporter (PGT), 15-hydroxyprostaglandin dehydrogenase (15-PGDH), EP3, β-catenin, ERK1/2, AKT and protein phosphatase 2 isoform beta of scaffold subunit A (PPP2R1B) was attempted. Only COX-1, 15-PGDH and β-catenin were reliably detected.

#### Confocal microscopy

Confocal images were captured using a Zeiss LSM 710 confocal microscope. Images were captured using either a × 20 objective (NA 0.8) or a × 63 oil immersion objective (NA 1.4). Pinhole size was set to 1, the pixel format was 1024 × 1024 and line averaging was employed to reduce noise.

### Determination of mucosal contents of PGE_2_ and prostaglandin E metabolites

Immediately upon extraction, biopsies were placed in 1 ml of Tris-buffered Ringer’s solution (pH 7.4) and snap frozen in liquid nitrogen. The Ringer’s solution was composed of (in mM) 20 Tris, 130 NaCl, 1 CaCl_2_, 2.5 K_2_HPO_2_ and 1 MgSO_4_ as well as protease inhibitor cocktail powder (one bottle dissolved in 100 ml Ringer’s solution), from Sigma-Aldrich (Schnelldorf, Germany, cat. no. P2714). In order to inhibit both synthesis and catabolism of PGE_2_, the Ringer’s solution additionally contained 13 μM of indomethacin from Sigma-Aldrich (Seelze, Germany) and 110 μM of the 15-PGDH-inhibitor 5-[[4-(ethoxycarbonyl)phenyl]azo]-2-hydroxy-benzeneacetic acid (Santa Cruz Biotechnology, Heidelberg, Germany, cat. no. 78028–01-0). 110 μM 15-PGDH inhibitor concentration was obtained by diluting a solution of 1 mg inhibitor dissolved in 33 μl DMSO to a ratio of 1 DMSO:833 Ringer. Samples were stored at − 80 °C. After a quick thaw, biopsies were retrieved and dabbed twice on Watman filter no 1 and weighed (range 5–15 mg) and suspended in 1 ml Ringer. Samples were then systematically pestel-squeezed to promote breakage of cell membranes and centrifuged for 30 s at 10,000 rpm in a Beckman microfuge to form a supernatant. Volumes of 50 μL supernatant were used for measuring PGE_2_ as well as the stable PGE_2_ metabolite PGE-M according to manufacturer’s instructions using ELISA kits from Cayman Chemical, Ann Arbor, MI, USA, cat. No. 514010 and 514,531. The ELISA results were adjusted for the weight of sample biopsy. Test for possible interference from the organic solvent DMSO was performed with up to double the maximal Ringer-added concentration of DMSO and demonstrated no shifts in the standard curves. Tests for general interference due to different dilutions of samples were performed and proved no need for adjustments.

## Results

### Patient characteristics

Twenty-seven patients were included. Fourteen patients in CRN group and 13 patients in control group. One additional patient was excluded from analysis due to histologically verified hyperplastic polyps, which are not classified as neoplasia. Median age was 64 years in CRN group and 62 years in control group. CRN group contained 7 (7/14) women versus 8 (8/13) in control group. Nine patients (9/14) had present CRN, while the 5 (5/14) remaining patients had previously been diagnosed with CRN. Three patients (3/14) had primary CRC. The remaining 11 (11/14) patients had tubular adenomas which were risk stratified as being high risk adenomas (*n* = 3/14), intermediate risk adenomas (*n* = 3/14) and low risk adenomas (*n* = 5/14) based on number, size and histology grade in accordance with the European guidelines for quality assurance in CRC screening and diagnosis [14]. Eight patients (8/14) from CRN group and 6 (6/13) patients from control group had one or more comorbidities such as anemia, diabetes, hypothyroidism, cardiovascular disease, asthma, osteoporosis, psoriasis, chronic dermatitis, allergic rhinitis and/or a psychiatric diagnosis. Two patients in CRN group had a history of primary prostate cancer without metastasis. In the control group 4 patients had a history of primary non-colorectal cancer without metastasis: Melanoma (*n* = 1/14), testis seminoma (*n* = 1/14), uterus cancer (*n* = 1/14) and breast cancer (*n* = 1/14). Nine patients (9/14) in CRN group and 10 (10/13) patients in control group received medications e.g. anti-thrombotics, angiotensin-converting-enzyme inhibitors, angiotensin-II-receptor antagonists, β-blockers, calcium-blockers, statins, diuretics, β2-agonists, anti-histamines, insulin, levothyroxine, proton pump inhibitors, laxantia, loperamid, anti-depressants, estrogen or bisphosphonates. There were no apparent differences between the CRN and control groups in use of medications nor in co-morbidity profiles. Level changes for *ERK*, *Akt*, *β-catenin* and *PPP2R1B* expression.

**Fig. 2:**
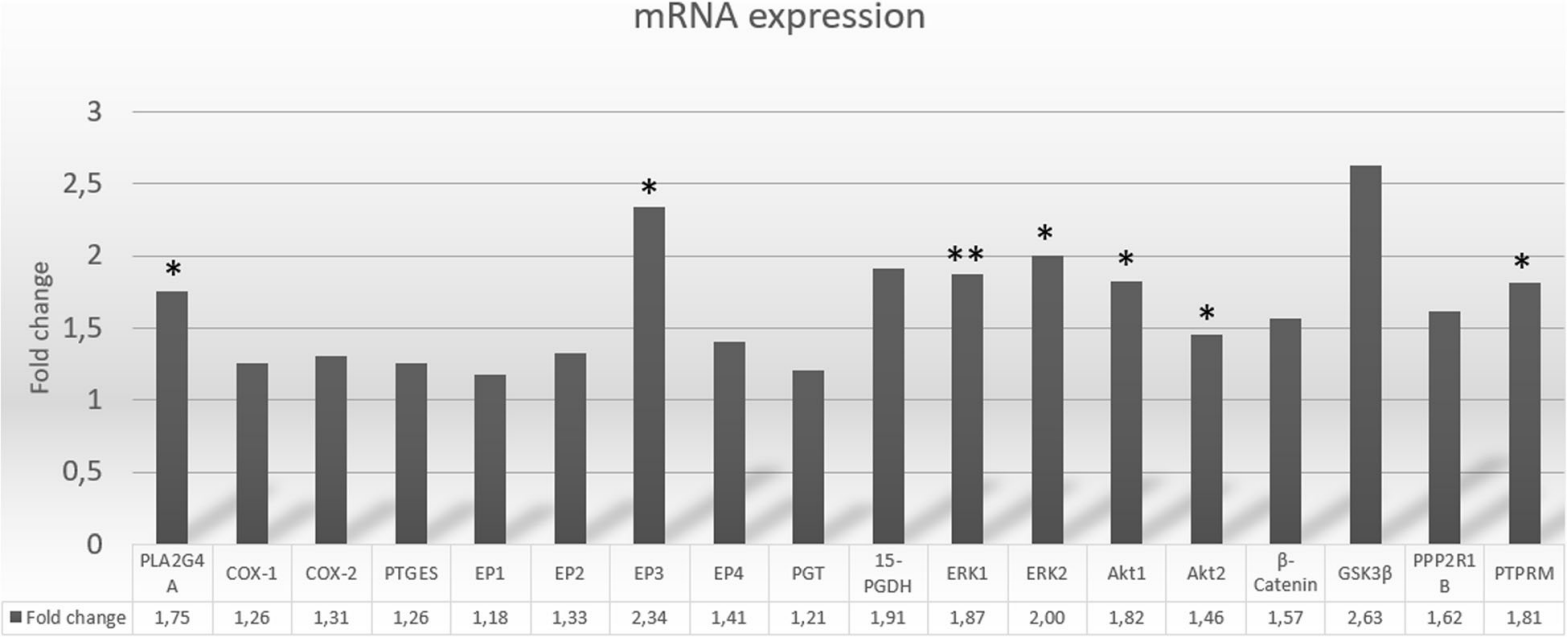
Fold change in mRNA expressions in normal appearing colonic mucosa from CRN patients compared to controls. CRN group: *N* = 14, Control group: *N* = 13. Data are expressed as fold change of mRNA expression in CRN group compared to control group

We screened colonic mucosa for a panel of 18 specific genes, all proven altered and involved in CRC development. All 18 targeted mRNAs were detected. Perturbed expression profile was observed in CRN patients with significantly higher mRNA-expressions measured for the following gene transcripts: *PLA2G4A*, *EP3*, *ERK1*, *ERK2*, *Akt1*, *Akt2* and *PPP2R1B*, Table 2 and Fig. 2.

**Table 2:** Difference in mRNA expression in CRN versus controls

	Control mean (±SEM)	CRN mean (±SEM)	Fold change	*P*
*PLA2G4A*	0.0063 (± 0.0008)	0.0110 (± 0.0017)	1.75	0.020*
*COX-1*	0.0054 (± 0.0007)	0.0069 (± 0.0009)	1.26	0.252
*COX-2*	0.0022 (± 0.0006)	0.0029 (± 0.0007)	1.31	0.490
*PTGES*	0.0008 (± 0.0001)	0.0010 (± 0.0002)	1.26	0.388
*EP1*	0.000029 (± 0.0000079)	0.000034 (± 0.0000064)	1.18	0.626
*EP2*	0.0013 (± 0.0002)	0.0018 (± 0.0003)	1.33	0.207
*EP3*	0.0011 (± 0.0002)	0.0026 (± 0.0005)	2.34	0.016*
*EP4*	0.0217 (± 0.0021)	0.0305 (± 0.0047)	1.41	0.096
*PGT*	0.0301 (± 0.0032)	0.0364 (± 0.0038)	1.21	0.217
*15-PGDH*	0.2283 (± 0.0572)	0.4350 (± 0.0921)	1.91	0.066
*ERK1*	0.0640 (± 0.0076)	0.1198 (± 0.0171)	1.87	0.007**
*ERK2*	0.0018 (± 0.0002)	0.0036 (± 0.0007)	2.00	0.020*
*Akt1*	0.0311 (± 0.0033)	0.0564 (± 0.0092)	1.82	0.020*
*Akt2*	0.0193 (± 0.0020)	0.0283 (± 0.0035)	1.46	0.041*
*β-Catenin*	0.1694 (± 0.0197)	0.2662 (± 0.0486)	1.57	0.082
*GSK3β*	0.0289 (± 0.0031)	0.0759 (± 0.0220)	2.63	0.054
*PPP2R1B*	0.0196 (± 0.0022)	0.0317 (± 0.0048)	1.62	0.030*
*PTPRM*	0.0079 (± 0.0011)	0.0142 (± 0.0029)	1.81	0.059

Control group: *N* = 13, CRN group: *N* = 14. Data are expressed as the mean (±SEM) and as fold change of mRNA expression in CRN group compared to control group. * = *p* < 0.05, ** = *p* < 0.01

For the CRN group we detected upregulation of *ERK1* (*p* = 0.007) and *ERK2* (*p* = 0.02) which are ubiquitous regulators of cellular proliferation, differentiation, survival and transformation. *ERK1* and *ERK2* were expressed 1.87 and 2.0 times higher, respectively, in CRN patients. *ERK1* expression was higher compared to *ERK2* in both patient groups. *Akt1* and *Akt2* were both significantly up-regulated in CRN group (*p* = 0.02 and *p* = 0.041, respectively). *β-catenin* was highly expressed in both groups and with substantial variability in CRN group. The phosphatase subunit *PPP2R1B*, which is considered a negative regulator of both ERK and Akt activation and a stimulator of Wnt/β-catenin signaling, was significantly up-regulated in CRN group (*p* = 0.03).

### Level changes for *PLA2G4A* and EP3

Analysis of entities in PGE_2_ metabolism by qPCR demonstrated expression of *PLA2G4A*, which encodes a major enzyme involved in arachidonic acid mobilization, was significantly increased in CRN group (*p* = 0.02). With respect to the EP-receptors, *EP4* had by far the highest expression, followed by *EP2* and *EP3*, while *EP1* showed the lowest expression. Only *EP3* was significantly up-regulated in CRN group compared to controls (*p* = 0.016). Of all investigated mRNAs, *15-PGDH* (the major enzyme involved in PGE_2_ degradation), showed the highest expression in both groups and 1.9-fold higher expression in CRN group compared to controls (*p* = 0.066). Expression of *COX-1* was higher than *COX-2* in both groups (CRN 2.4-fold; controls 2.5-fold), while none of the two COX enzymes were significantly altered between patient groups. When Bonferroni correction was applied none of the 18 investigated genes reached the required significance level of *p* < 0.0028.

### Trend towards increase in β-catenin, COX-1, COX-2 and ERK1 protein expression

**Fig. 3:**
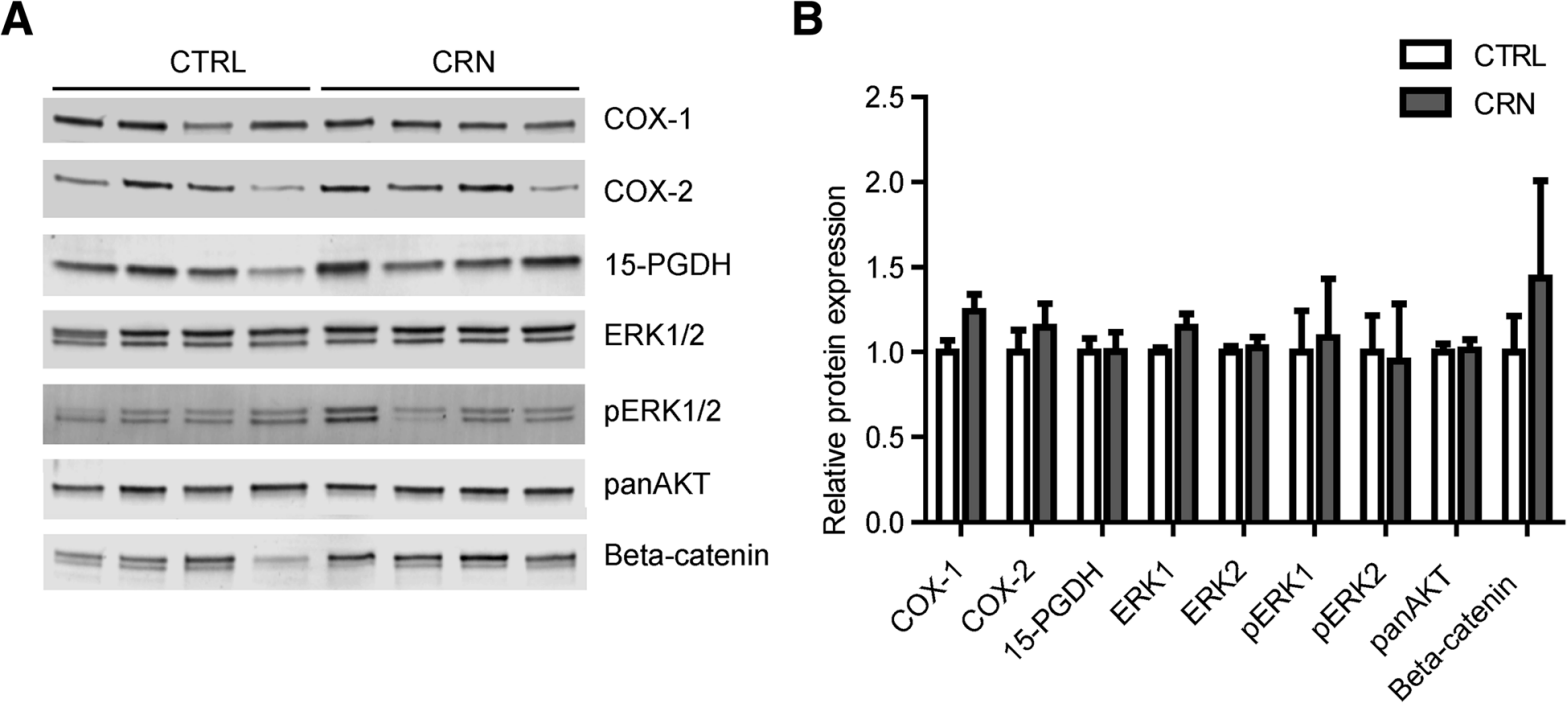
Protein expression. **a** Representative Western blot images for seven different targets, indicated to the right of bands. CTRL: samples originate from control group, CRN: samples originate from neoplasia group. **b** Quantification of band intensities for nine different target proteins. REVERT™ Total Protein Stain was used as internal loading control (please see materials and methods section). The expression level of each target protein was normalized to the mean expression level in the control group which was set to 1. Data are expressed as the mean ± SEM

To determine whether the changed mRNA expression profile observed in CRN patients was associated with changes in the protein expression level of these targets, we next performed Western blot analysis on colonic biopsies from CRN patients and controls. We successfully detected COX-1, COX-2, 15-PGDH, ERK1/2 and the active phosphorylated ERK1/2 (pERK1/2) proteins as well as AKT (using an antibody that detects AKT1–3) and β-catenin. We analyzed the protein expression level for each of these targets in the control group (12 biopsies) and compared them to the expression levels in the CRN group (12 biopsies). Relative protein expressions for the detected target proteins are shown in Fig. 3. The Western blot results suggest a trend towards a moderate increase in β-catenin as well as minor increases in COX-1, COX-2 and ERK1. The observed changes were not, however, statistically significant.

### Localization of COX-1, 15-PGDH and β-catenin

**Fig. 4:**
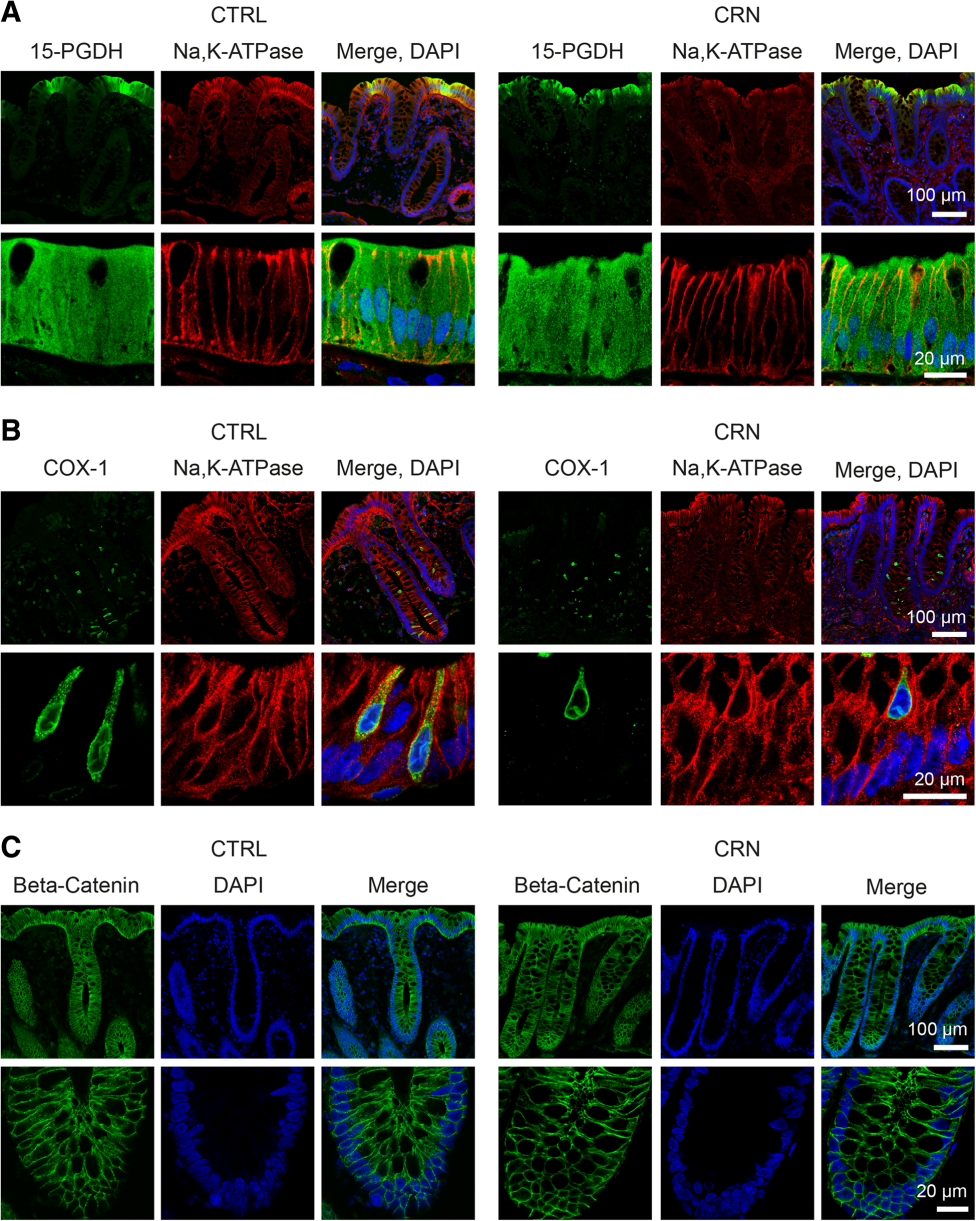
Immunohistochemical analysis of 15-PGDH, COX-1 and β-catenin. Representative confocal images demonstrating the cellular and subcellular localization of 15-PGDH (**a**) and COX-1 (**b**) and β-catenin (**c**) in human colonic biopsies. Staining for Na-K-ATPase and DAPI was included to locate the plasma membrane and nuclei, respectively. To the left: CTRL; Control group. To the right: CRN; colorectal neoplasia group

We next sought to investigate the localization of target proteins by immunohistochemistry. We attempted to detect cPLA2A, COX-1, COX-2, EP3, PGT, 15-PGDH, ERK1/2, AKT, β-catenin and PPP2R1B in colonic biopsies from CRN patients and controls. The targets were selected to map localizations of proteins involved in different aspects of the PGE_2_ pathway and to study any differences in protein localizations and/or possible expressions in CRN patients compared to controls. Possibly due to inadequate (human) specificity of the tested antibodies or paucity of target proteins, we failed to reliably detect cPLA2A, COX-2, PGT, EP3, ERK1/2, AKT and PPP2R1B in IHC. Only COX-1, 15-PGDH and β-catenin were detected. The results are shown in Fig. 4. 15-PGDH displayed strong cytoplasmic staining in epithelial cells at the crypt apex. COX-1 was selectively expressed in a small subset of cells in the epithelial cell layer where the enzyme displayed a reticular, intracellular localization associated with the perinuclear area. We believe that these COX-1 expressing cells represent epithelial tuft cells [15, 16]. β-catenin showed expression throughout the epithelial layer and was mainly associated with lateral membranes of the epithelial cells. No apparent differences in protein localization were observed between CRN and control groups.

### Indication of increased prostaglandin E2 content in mucosa of CRN patients

**Fig. 5:**
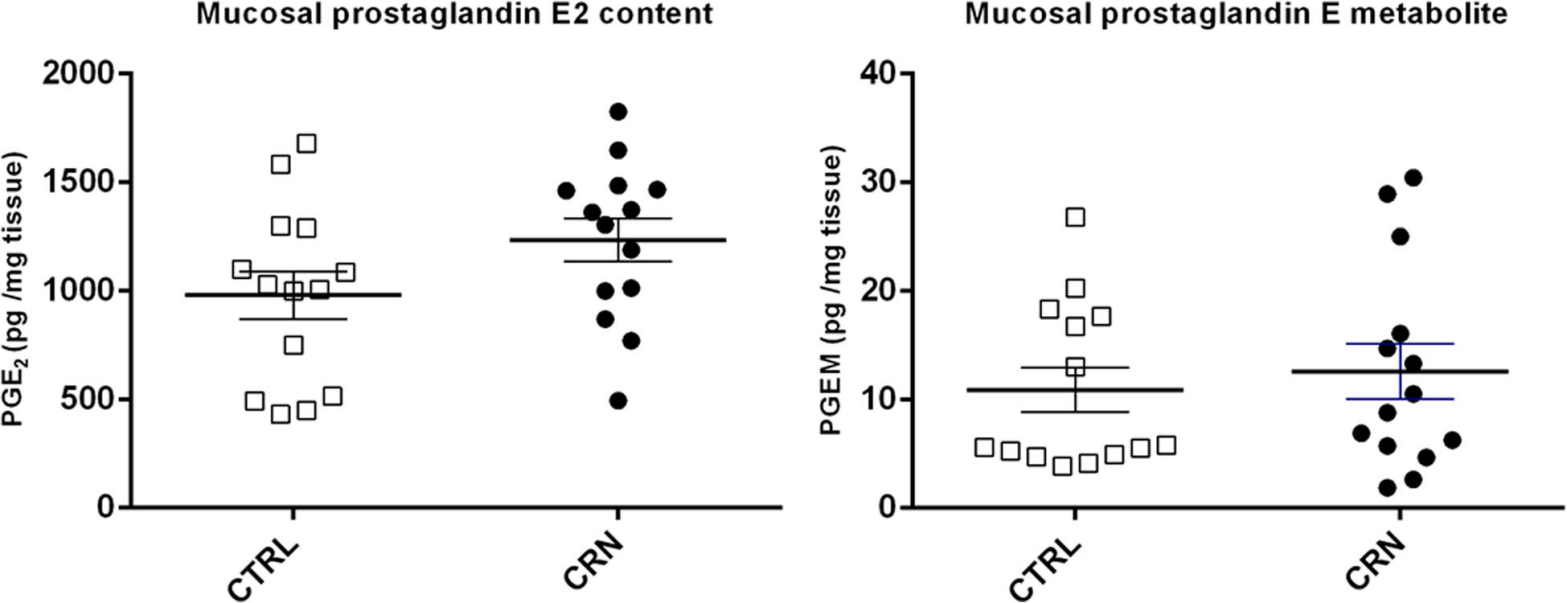
Mucosal prostaglandin E2 contents. On the left: Mucosal prostaglandin E2 concentrations. On the right: Mucosal concentration of prostaglandin E metabolites. Each mark represents one study patient. White squares = CTRL, control group (*N* = 14), Black dots = CRN group (*N* = 14)

Mean mucosal PGE_2_ content (pg/mg tissue) was 1234 (± 98) in CRN group and 980 (± 109) in control group, *p* = 0.095, Fig. 5. Translated to tissue concentrations, the values come to 3.50 μM in CRN and 2.78 μM in control group. Mean mucosal PGE_2_ metabolite content (pg/mg tissue) was 12.58 (± 2.54) in CRN group versus 10.90 (± 2.05) in control group.

## Discussion

Normal appearing, tumor-remote colonic mucosa in CRN patients has so far been scarcely explored. Here we document significant up-regulation of mRNA expression for the *ERK1*, *ERK2*, *Akt1*, *Akt2*, *PLA2G4A*, the prostanoid receptor *EP3* as well as for *PPP2R1B* in normal appearing colonic mucosa from patients with CRN, Table 2. The range in fold change of these gene expressions was 1.46–2.34. The fact that our observed changes in gene expression were somewhat modest compared to changes reported in cancer tissues was to be expected since we examined normal appearing colon mucosa distant from neoplastic tissue. Furthermore, CRN group encompassed a wide range of stages of CRN and both patients with current CRN as wells as patients with a history of CRN. Since we perceive CRN development as a spectrum from low-grade adenomas to CRC, all CRN data were pooled into one group, tacitly acknowledging that different stages of CRN are likely to contribute differentially to the observed alterations of gene expression. Thus, it was a heterogenous CRN group with a limited number of observations and hence the observed changes between groups were not strong enough to reach the required significance level of a Bonferroni correction. However, our results point towards perturbed gene expression in the normal colon mucosa of CRN patients. These alterations in mRNA expression did not translate into significant alterations at protein level, Fig. 3. Although, the mRNA expressions of the above stated gene transcripts have previously been shown perturbed in CRC affected mucosa, we do not know the precise biological impact of the altered gene expressions in the normal appearing mucosa.

### ERK and Akt signaling pathways

We observed significant up-regulation of mRNA expression for *ERK1, ERK2, Akt1 and Akt2* in normal appearing mucosa of CRN patients. Contrary, examining protein abundance, we found a marginal increase of ERK1 (*p* = 0.09) in CRN-group, while phospho-ERK1, ERK2 and panAkt were similar between groups. One might argue that protein expression, especially for phosphorylated proteins, is a more direct marker for cell function compared to mRNA. However, Western blot measurements of protein expression are far less accurate than the qPCR method for measuring mRNA. The observed up-regulation of both *ERK* and *Akt* mRNAs are quite interesting findings given the fact that ERK/MAPK and PI3K/Akt pathways are well established important pathways in regulating proliferation and human carcinoma survival [17, 18]. *ERK1* and *ERK2* are ubiquitous regulators of multiple cellular processes and dysregulated nuclear accumulation of activated ERKs (pERK) can lead to genomic instability and subsequent CRN progression [19–23]. It is difficult to assign a precise role of ERK signaling in human carcinogenesis due to its complexity and dependence on signaling intensity. Still, ERKs may play oncogenic and/or tumor suppressing roles in normal appearing colonic mucosa [24–26]. Our findings of markedly lifted expression levels for *ERK1* points to this kinase as a possible useful predictive biomarker for CRN development. In addition to *ERK* and *Akt*, we found enhanced mRNA expression of the structural subunit Aβ, *PPP2R1B*, which is an isoform of the scaffold subunit of protein phosphatase 2A (PP2A). PP2A is considered a tumor suppressor and a negative regulator of both ERK and Akt activation and stimulator of Wnt/β-catenin signaling [27, 28]. In the present study, *PPP2R1B* mRNA was up-regulated in CRN group and we speculate that this is a compensatory up-regulation to counteract up-regulation of Akt and ERK.

### Prostaglandin E2 signaling

#### Prostaglandin E2 synthesis and mucosal content

As mentioned *PLA2G4A* mRNA expression is up-regulated in normal appearing colonic mucosa from CRN patients, Table 2. The *PLA2G4A* gene encodes the enzyme cPLA2A which is a key enzyme involved in arachidonic acid mobilization and upstream release of many lipid mediators including lysophospholipids, prostaglandins, leukotrienes and lipoxins [29, 30]. Understanding how the cPLA2A enzyme regulates tumorigenesis is hampered by the entwined effects of its many interacting products of downstream eicosanoid mediators. Meanwhile, our result of up-regulated *PLA2G4A* could indicate an increased substrate concentration for eicosanoid generation. Thus, despite we did not observe up-regulation of *COX-1* and *-2* enzyme mRNA, a higher substrate level may yield a higher PGE_2_-concentration. This corroborates with previous reports demonstrating higher sensitivity to indomethacin in CRN patients versus controls [3, 16, 31]. In terms of the resulting mucosal content of active PGE_2_, our assay indicates that the mean concentration is lifted (26%) in CRN patients. The observed mucosal PGE_2_ content in this study may be transformed into tissue concentrations of 3.50 and 2.78 μM for CRN and control patients, respectively. Based on these findings and our finding of a high-affinity EP4 subtype receptor (estimated EC_50_ of 10 nM) in similar patient mucosal tissue samples (S. Kjaergaard et al., unpublished; U.R. Feddersen et al., unpublished), we assume that the majority of PGE_2_ is to be found intracellularly and that the extracellular mucosal concentrations are 3 orders of magnitude lower. Taken together, findings suggest presence of a mild chronic inflammation in endoscopically normal appearing mucosa of CRN patients. A similar 27% marginal increase (*p* = 0.09) in mean colonic mucosal PGE_2_ concentration was reported by Krishnan et al. for patients treated for CRN [32].

### Prostaglandin E2 receptors

Investigating EP receptors, we found *EP3* to be up-regulated in CRN patients, Table 2. Interestingly, a study of paired colonic normal and tumor tissues from CRN patients, reported the tumor tissue had downregulated expression of *EP3* mRNA [33]. Of note, the EP3 receptor is unique among EP receptor subtypes, in that there are multiple isoforms generated through alternative mRNA splicing in the carboxyl tail of the *EP3* gene resulting in isoform specific differences in G-protein coupling and signaling [34]. Our *EP3* qPCR-primer did not differentiate between EP3 isoforms. Nonetheless, the major EP3 isoform is thought to couple to an inhibitory G protein (Gi), and hence the major outcome of PGE_2_-EP3 receptor signaling is inhibition of adenylate cyclase and activation of the ERK/MAPK pathway [35]. Thus, the role of the EP3 receptor in tumorigenesis seems to be multifaceted and isoform-dependent. As for cPLA2A, azoxymethane-induced colon cancer development is enhanced in *EP3* receptor knockout mice, suggesting an antitumorigenic function for the EP3 receptor [33, 36]. Further studies of the mRNA and protein expressions of individual EP3 isoforms in normal appearing colonic mucosa from humans are warranted.

### Prostaglandin E2 influx and degradation

In terms of cellular PGE_2_ influx and subsequent elimination we found no change in mRNA expressions of the major and specific PGE_2_ influx transporter *PGT* in CRN patients. The expression of *PGT*, an organic anion polypeptide transporter (OATP) has been reported downregulated in CRC tissue [37]. However, other specific PGE_2_ OATP-transporters, potentially involved in regulatory removal of PGE_2_, were not investigated in the present study. However, our group reported compensatory increase in the level of two OATP PGE_2_ influx transporters, OATP2B1 and OATP4A1, located in the basolateral membrane of human colonic epithelia from CRN patients [38]. Since lowered expression of the PGE_2_ inactivating enzyme, *15-PGDH*, has been observed in cancer cells [39], we hypothesized a decrease in *15-PGDH* expression in CRN patients compared to controls. Meanwhile, this hypothesis was not supported by a nearly 2-fold (*p* = 0.066) increase in *15-PGDH* mRNA expression in CRN patients. The mechanism(s) for the observed elevation of *15-PGDH* mRNA expression in the normal appearing colonic mucosa of CRN patients is unresolved. We speculate that a negative feedback balance between increased PGE_2_ production and subsequent increase in removal/inactivation is present in the normal appearing mucosa of CRN patients.

### Is our finding of perturbed mRNA expression a CRN predisposition?

Our suggestions of perturbed mRNA expressions as possible predisposing factors for the individual development of CRN could also be explained by the extant tumor tissue per se. Thus, at play could be local paracrine inducers or more systemic neuro-, endo- or immunocrine signaling from tumor to neighboring colonic mucosal areas. Yet, another pathway might be special cancer-inducing environmental stimulants as products of food digestion or microbial activity affecting gene expression in non-tumor mucosa. A conclusive settlement of this question between inborn predisposing constitutions versus tumor activity or environmental factors in normal appearing epithelium will require larger population studies. Regardless of the underlying mechanisms, our observation of up-regulation of several genes in normal appearing colonic mucosa suggests that normal appearing mucosa of CRN patients differs from non-CRN patients at a molecular level. Since up-regulated genes in this study have been found perturbed in CRC studies on colonic biopsies of affected mucosa, they could indicate possible predispositions for CRC development. The exploratory nature of our relatively small study cohort points to a need for further confirmation in larger prospective studies in order to determine if the observed aberrant marker genes may be useful predictive biomarkers.

## Conclusion

We observed significant up-regulation of *ERK1*, *ERK2*, *Akt1*, *Akt2*, *PLA2G4A*, prostanoid receptor *EP3* and phosphatase scaffold subunit *PPP2R1B* mRNA expression in normal appearing colonic mucosa of patients with CRN. Accordingly, normal appearing mucosa of CRN patients differs from non-CRN patients at a molecular level. Most notably, mRNA expression of *ERK1* was lifted with high significance of *p* = 0.007 and may therefore be considered a potential candidate gene as predictive biomarker for developing CRN. Our observations need to be validated in larger prospective studies.

## Data Availability

The datasets used and/or analyzed during the current study are available from the corresponding author on reasonable request.

## Abbreviations

15-PGDH: 15-hydroxyprostaglandin dehydrogenase
Akt1 and 2: Subtypes 1 and 2 of the Akt protein kinase (protein kinase B)
APC: Adenomatous polyposis coli
COX-1 and COX-2: Cyclooxygenase subtypes 1 and 2
cPLA2A: Cytosplamic phospholipase A2 alpha
CRC: Colorectal cancer
CRN: Colorectal neoplasia
EP1, EP2, EP3 and EP4: PGE_2_ receptor subtypes EP 1–4
ERK1 and 2: Subtypes 1 and 2 of the extracellular signal-regulated kinase
GPCR: G-protein coupled receptor
GSK3β: Glycogen synthase kinase 3 beta
MAPK: Mitogen activated protein kinase
mTOR: Mammalian target of rapamycin
OATP: Organic anion polypeptide transporter
PGE_2_: Prostaglandin E2
PGH_2_: Prostaglandin H2
PGT: Prostaglandin transporter
PI3K: Phosphoinositide 3-kinase
PIK3CA: CA phosphoinositide-3-kinase gene
PIP3: Phosphatidyl inositol triphosphate
PLA2G4A: Phospholipase A2 group IVA
PP2A: Protein phosphatase 2A
PPP2R1B: Isoform beta of scaffold subunit A for protein phosphatase 2
PTEN: Phosphatase and tensin homolog
RTK: Receptor tyrosin kinase
